# Accurate Segmentation of Nuclear Regions with Multi-Organ Histopathology Images Using Artificial Intelligence for Cancer Diagnosis in Personalized Medicine

**DOI:** 10.3390/jpm11060515

**Published:** 2021-06-04

**Authors:** Tahir Mahmood, Muhammad Owais, Kyoung Jun Noh, Hyo Sik Yoon, Ja Hyung Koo, Adnan Haider, Haseeb Sultan, Kang Ryoung Park

**Affiliations:** Division of Electronics and Electrical Engineering, Dongguk University, 30 Pildong-ro 1-gil, Jung-gu, Seoul 04620, Korea; tahirmahmood.cs@gmail.com (T.M.); malikowais266@gmail.com (M.O.); nohkyungjun@dongguk.edu (K.J.N.); yoonhs@dongguk.edu (H.S.Y.); koo6190@dongguk.edu (J.H.K.); adnanhaider@dgu.ac.kr (A.H.); haseebsltn@gmail.com (H.S.)

**Keywords:** multi-organ histopathology images, triple-negative breast cancer, The Cancer Genome Atlas, artificial intelligence, nuclear segmentation, stain normalization, cancer grading and prognosis

## Abstract

Accurate nuclear segmentation in histopathology images plays a key role in digital pathology. It is considered a prerequisite for the determination of cell phenotype, nuclear morphometrics, cell classification, and the grading and prognosis of cancer. However, it is a very challenging task because of the different types of nuclei, large intraclass variations, and diverse cell morphologies. Consequently, the manual inspection of such images under high-resolution microscopes is tedious and time-consuming. Alternatively, artificial intelligence (AI)-based automated techniques, which are fast and robust, and require less human effort, can be used. Recently, several AI-based nuclear segmentation techniques have been proposed. They have shown a significant performance improvement for this task, but there is room for further improvement. Thus, we propose an AI-based nuclear segmentation technique in which we adopt a new nuclear segmentation network empowered by residual skip connections to address this issue. Experiments were performed on two publicly available datasets: (1) The Cancer Genome Atlas (TCGA), and (2) Triple-Negative Breast Cancer (TNBC). The results show that our proposed technique achieves an aggregated Jaccard index (AJI) of 0.6794, Dice coefficient of 0.8084, and F1-measure of 0.8547 on TCGA dataset, and an AJI of 0.7332, Dice coefficient of 0.8441, precision of 0.8352, recall of 0.8306, and F1-measure of 0.8329 on the TNBC dataset. These values are higher than those of the state-of-the-art methods.

## 1. Introduction

A nucleus is a highly specialized organelle that serves as the information processing and administrative center of a cell. It has been studied to determine the cell and tissue phenotypes, cellular processes, and cell populations [1]. It can also be used to determine mitosis and the level of nuclear pleomorphism, which are used for the grading and prognosis of cancer [2]. Moreover, nuclear morphology and features, such as density, size, and shape, are helpful for the assessment of treatment effectiveness [3,4]. Nuclear segmentation can enable the extraction of high-quality features for nuclear morphometric and other analyses. Kumar et al. proposed a method [5] for prostate cancer recurrence prediction based on nuclear segmentation. Zhao et al. proposed a selective-edge-enhancement-based nuclear segmentation method [6] for cervical smear images, which play a crucial role in cervical cancer detection. Quantification of protein expression and the study of cell function can also be done after nuclear segmentation. Gharipour et al. proposed a method [7] using a region-based active contour model in a variational level set formulation for fluorescence microscopy images. Breast cancer detection from cytological images is standard practice, and nuclear segmentation plays a key role as it allows observing breast cancer malignancy [8]. George et al. proposed an automated nuclear segmentation method [8] in breast cancer histopathology images for diagnosis and prognosis of breast cancer.

**Figure 1:**
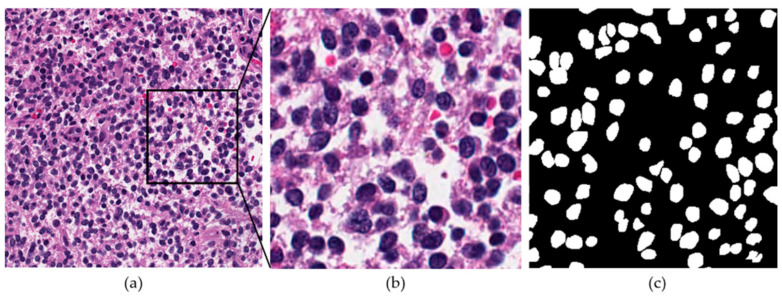
Sample histopathology image for nuclear segmentation: (**a**) histopathology image, (**b**) zoomed image, and (**c**) ground-truth image for (**b**). Blue regions in (**a**,**b**) and white areas in (**c**) represent nuclei, which need to be segmented.

Histopathology images are widely used to assess the grade and prognosis of cancer [9]. Biopsies or surgical specimens are studied under high-resolution microscopes by pathologists after being processed through a staining procedure. Despite the standardization of staining procedures, there remains a significant variation in the color, intensity, and morphological features of images. Consequently, this procedure has several limitations. For example, the analysis of multiple stain slides per patient is tiresome, which can affect the pathological diagnostic performance. Consequently, digital pathology is widely used to address these issues. Digital pathology has become increasingly popular in the past decade due to improvements in computer vision techniques. Today, whole-slide images (WSIs) can be easily acquired, stored, and processed using dedicated scanners and software. Moreover, advanced artificial intelligence (AI)-based techniques that automatically analyze and quantify information from tissue slides have been developed. The task of nuclear segmentation has also been addressed using AI-based automated methods. However, this task is very challenging because different types of nuclei exist depending on the organ type. Furthermore, intra- and interclass variations in the morphological appearance of nuclei exist. Figure 1 presents a sample histopathology image to demonstrate the complexity of the problem.

Recently, AI-based methods have been developed to address various problems [10,11,12]. Accordingly, AI has been adopted in digital pathology for various diagnostic systems [13,14] because AI-based solutions are robust and fast, and require less human effort. Owing to the importance of nuclear segmentation in digital pathology, researchers have proposed several methods based on either conventional image processing or AI-based techniques. Conventional image processing methods are mainly based on algorithms such as Otsu’s thresholding [15], the watershed algorithm [16], and gradient vector flow [17]. However, these methods lack robustness and require parameter settings. Consequently, AI-based methods have been adopted to overcome these problems. These methods have shown a significant performance improvement for the nuclear segmentation task, but there is room for further improvement. Therefore, we propose a nuclear segmentation method based on a novel residual-skip-connections-based segmentation network for nuclei (R-SNN). Two publicly available datasets—The Cancer Genome Atlas (TCGA) and Triple-Negative Breast Cancer (TNBC)—are used in the experiments. These datasets comprise images from various organs, including the brain, breast, kidney, liver, prostate, bladder, colon, stomach, and lungs. Experimental results show that our proposed technique is superior to the state-of-the-art methods.

Our study is novel in five ways compared with previous works.

- In our proposed method, full-size patches of 1000 × 1000 pixels for TCGA dataset and 512 × 512 pixels for the TNBC dataset are processed without converting them into sub-patches, whereas existing methods converts full-size patches into sub-patches before performing segmentation. The proposed method exhibited good segmentation performance without the additional requirements of sub-patch conversion. In addition, our method does not require postprocessing, unlike other nuclear segmentation techniques.
- The performance of nuclear segmentation is improved by maintaining high-frequency information owing to spatial information transfer from the encoder to the decoder through residual connectivity.
- With the specified design, we reduce the number of convolution layers, and the proposed R-SNN utilizes fewer trainable parameters.
- The training of the network is fast, and the network converges rapidly in only 30 epochs (27,210 iterations) on average, owing to the residual connections and reduced structure of the network.
- As shown in [18], our trained models and codes are available on request for research purposes.

The remainder of this article is organized as follows: Section 2 describes the related work. Section 3 presents the proposed method. Section 4 presents the experiments and performance analysis. Section 5 provides the discussion. Section 6 presents the conclusion.

## 2. Related Works

Although studies on digital image analysis have been conducted previously [19], recent developments in digital pathology have triggered a surge in the development of AI-based methods. The nuclear segmentation task is addressed by using either conventional handcrafted-feature-based or deep-feature-based methods.

### 2.1. Handcrafted-Feature-Based Methods

Conventional handcrafted-feature-based techniques for nuclear segmentation are mostly based on watershed segmentation [20], mathematical morphology, graph-based segmentation, color-based thresholding, active contours, and their variants. Yang et al. proposed a nuclear segmentation technique for time-lapse microscopy images by using marker-controlled watershed segmentation [21]. Context information among the neighboring frames was utilized for the over-segmented and under-segmented cells. Moreover, a combination of mean shift and the Kalman filter was used for tracking purposes. Cosatto et al. [22] proposed a technique based on the Hough transform [23] and active contour model. The nuclear pleomorphism was predicted from the segmented nuclei. Ali et al. [24] proposed a technique based on an active contour model that integrates the region, boundary, and shape information. They proved that their technique could be used for the segmentation of nuclei, lymphocytes, and glands in histopathology images. Huang et al. presented a technique based on marker-controlled watershed segmentation, followed by refinement through a snake model, and finally classification using a support vector machine (SVM)-based decision graph classifier [20]. The major limitation of nuclear segmentation techniques based on handcrafted features is their sensitivity to the parameter settings and the specific types of nuclei structures. Therefore, a generalized and robust nuclear segmentation technique is required.

### 2.2. Deep-Feature-Based Methods

In recent years, researchers have proposed different techniques based on deep learning. In conventional machine-learning techniques, feature processing is used for the collection of optimal features and the training of machine-learning models. Features such as shape, color, texture, color histogram, Laplacian of Gaussian, and gradients have been used [25,26]. In contrast, deep-learning techniques are based on automatic feature extraction, and they have been used to develop robust, fast, and high-performance models. Different types of deep-learning models have been adopted. One such example is the mask-region convolutional neural network (RCNN) [27], which has been used in other studies for nuclear segmentation and achieved a good performance [28]. A fully convolutional network (FCN) has also been used for this task. FCN is a well-known segmentation network that achieves good results but requires many learnable parameters, which need to be reduced. Kumar et al. provided a nuclear segmentation dataset and proposed a simple nuclear segmentation technique by considering nuclear segmentation as a three-class problem [29]. They compared their results with those of CellProfiler (CP) [30] and Fiji (ImageJ) [31] and proved that the performance of a simple CNN-based network is better than that of CP and Fiji. Similarly, Naylor et al. [32] provided another dataset and performed experiments on three well-known networks, namely, PangNet [33], FCN [34], and DeconvNet [35], for the nuclear segmentation. In addition, they used the ensemble classification model, which showed high accuracy. Kang et al. proposed a technique based on two-stage learning and deep layer aggregation (DLA) [36]. They considered nuclear segmentation as a three-class problem by considering the boundaries of the nuclei as the third class. Their method comprised two stages, which consisted of a U-Net empowered by the DLA. Zhou et al. used spatial and texture dependencies between the nuclei and the contour in their proposed method [37]. A contour-aware informative aggregation network (CIA-Net) was proposed for nuclear segmentation in which the information was aggregated at multiple levels using two task-specific decoders. Losses were modulated using a novel smooth truncated loss. U-Net was also used for the nuclear segmentation task. U-Net is a convolutional network designed for medical applications. Mahbod et al. [38] proposed a technique containing two sequential stages in which nuclei were separated from the background using a U-Net-based classification network in stage 1, followed by the generation of a distance map using regression U-Net in stage 2. Zeng et al. [39] proposed a technique based on U-Net empowered by inception modules. Chidester et al. proposed a technique based on rotation-equivariant convolutional layers in a U-Net architecture [40]. Although most previous studies showed good accuracy, there is room for further performance improvement. In addition, most of these methods require additional postprocessing. In previous research [41], the authors proposed a stain normalization method of whole-slide images in histopathology, but they did not deal with nuclear segmentation with the stain-normalized image.

Although it did not deal with nuclear segmentation, a previous study proposed OR-Skip-Net [42], to which our R-SNN is different as described below. In terms of applications, R-SNN is an end-to-end semantic segmentation network for nuclear segmentation, whereas OR-Skip-Net is an end-to-end semantic segmentation network for skin and gland segmentation. In terms of the number of convolution layers, R-SNN has 20 convolution layers. The encoder and decoder use 10 convolution layers each, whereas OR-Skip-Net has 16 convolution layers with the encoder and decoder using eight convolution layers each. R-SNN has four encoder and four decoder blocks, whereby the first two blocks of encoder and decoder have two convolution layers while the other two blocks have three convolution layers. However, OR-Skip-Net also has four blocks, but each block of the encoder and decoder has two convolutional layers. R-SNN skip connections use identity mapping for the addition of residual connections, whereas OR-Skip-Net skip connections use non-identity mapping for the addition of residual connections. Lastly, R-SNN has a total of 15,279,174 trainable parameters, whereas OR-Skip-Net has a total of 9,718,786 trainable parameters.

Considering the limitations of previous research, we propose a nuclear segmentation method based on the novel R-SNN. Table 1 presents a comparison between the existing methods and our nuclear segmentation technique.

## 3. Proposed Method

### 3.1. Overview of the Proposed Architecture

**Figure 2:**
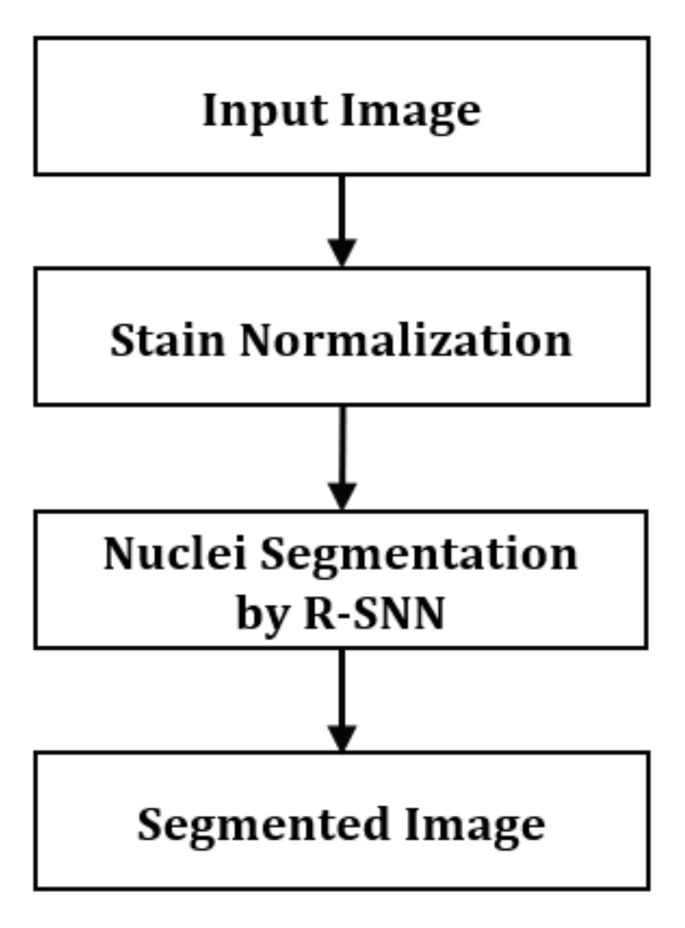
Overview of the proposed technique.

The proposed method has two main stages: stain normalization and nuclear segmentation, as shown in Figure 2. In the first stage, a histopathology image is stain-normalized to balance the color and intensity variation. Subsequently, it is used as an input to the R-SNN which outputs a segmented image.

### 3.2. Preprocessing by Stain Normalization

Stain normalization techniques aim to reduce the variations in color and intensity of histopathology images by generating images with a standard appearance [41]. In our proposed technique, we adopted the stain normalization technique proposed by Macenko et al. [43] in which optical density (OD) and color deconvolution schemes are used. The RGB color values of an input image are converted into OD values. OD is the log ratio of the incident light image (I_i_) and transmitted light image (I_t_) and is represented by Equation (1).

OD = Log_10_ (I_i_/I_t_), (1)

**Figure 3:**
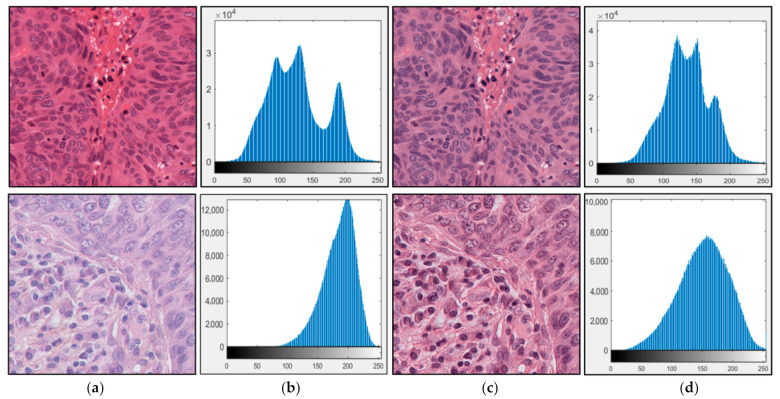
Sample images after stain normalization: (**a**) original image, (**b**) histogram of (**a**), (**c**) normalized image, and (**d**) histogram of (**c**). The upper and lower images represent TCGA and TNBC datasets, respectively. In (**b**,**d**), horizontal and vertical axes represent pixel value and the number of pixels, respectively.

In Equation (1), I_i_ represents the input image, whereas I_t_ is the transmitted light image. In our proposed technique we adopted I_t_ = 240 for both training and testing images on the basis of previous research [43]. The transformation of RGB values into OD values results in a space where the linear combination of stains would result in a linear combination of OD values. Then, all pixels with 0 or low OD values are removed by using a threshold called β. We used β = 0.15 on the basis of previous research [43]. After this, singular value decomposition (SVD) is performed on the OD tuples, and a plane is created from the SVD directions. The OD values are projected onto the plane and normalized to a unit length. The angle of each point is calculated with respect to the first SVD direction and, then, the robust extremes of the angle are calculated. They are then converted back to OD to obtain the optimal stain vector. Figure 3 shows the images obtained using the stain normalization method. This figure shows that the variations in color and intensity are reduced in the normalized images compared with those in the original images. In addition, we show pre- and post-normalization image histograms for quantitative comparison. As shown in Figure 3, we confirm that the post-normalization image histograms are more closely related to a Gaussian distribution with the mean value close to the median pixel value (127) than the pre-normalization image histograms.

### 3.3. Architecture of the Proposed R-SNN

The proposed R-SNN is an end-to-end encoder–decoder semantic segmentation network in which the input image is first downsampled by passing it through multiple deep-learning convolution and pooling layers in the encoder part and then upsampled to the original size by the decoder part. The convolution layers create a feature map that represents the significant features in the input image, and these features are used for the training of the network. The pooling layer is responsible for the reduction in the number of parameters and computation time by downsampling the input image and feature map. The number and design of layers are important because information may be lost during extraction, which results in the performance degradation of the network [42]. In the nuclear segmentation problem, the regions of interest (ROIs) are small with diverse morphological features. Therefore, the design of a semantic segmentation network is very challenging because, if a shallow network with very few layers is developed, the model may not be robust. In contrast, if a deep model having several layers is developed, semantic information may be lost due to successive convolution and pooling operations, which can negatively affect the performance of the model. Therefore, the proposed model is developed by considering all the aforementioned aspects.

**Figure 4:**
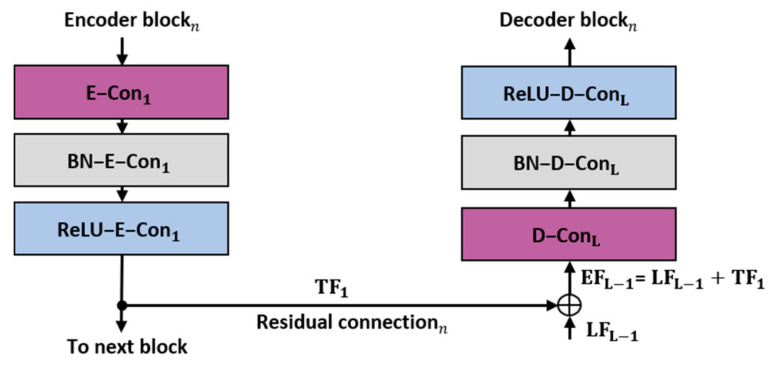
Schematic of residual connectivity used in the R-SNN. E-Con_1_, BN-E-Con_1_, and ReLU-E-Con_1_ indicate the convolution layer of the encoder, batch normalization, and rectified linear unit of the first layer, respectively. D-Con_L_, BN-D-Con_L_, and ReLU-D-Con_L_ indicate the convolution layer of the decoder, batch normalization, and rectified linear unit of the L-th layer, respectively.

**Figure 5:**
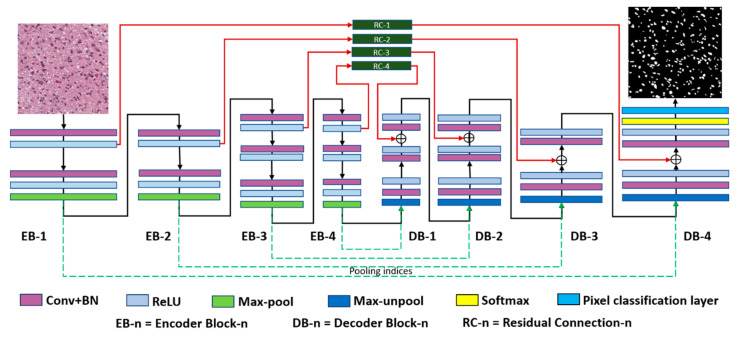
Proposed R-SNN.

The residual connectivity proposed in the residual network (ResNet) reduces the loss of information and resolves the vanishing gradient problem [44]. The proposed R-SNN uses residual connectivity to empower the feature passing through the network. Unlike conventional ResNet [44], which connects the convolutional layers of the current block via a residual connection, the proposed R-SNN directly connects each encoder block to the corresponding decoder block using residual skip connections. As shown in Figure 4 and Figure 5, these skip connections begin after the first convolutional layer of each encoder block and terminate before the last convolutional layer of each decoder block. Specifically, as shown in Figure 4, the convolutional layer of the decoder block produces the local features **LF_L-i_**, which are combined with the transferred features **TF** from the encoder through element-wise addition, resulting in the enhanced features **EF_L-I_,** given by Equation (2).

**EF_L-i_** = **LF_L-i_ + TF,** (2)

where **EF**, **LF**, and **TF** represent the enhanced, local, and transferred features, and i = 1 represents the second last layer of each block, respectively. The subscript L denotes the L-th layer of each block, from which the features are extracted. The performance of the proposed model is enhanced through residual connectivity.

The second important aspect of our proposed R-SNN is the use of fewer layers in our model and the confinement of the feature map size at the last convolution block to 31 × 31. The feature map from the last convolution block is important when dealing with tiny object classification and segmentation tasks. There is a tradeoff between the loss of semantic information and the feature map size. In deep networks, more concentrated and filtered information is obtained; however, information that can play an important role in the segmentation of tiny ROIs may be lost. The vanishing gradient problem may also occur. In contrast, shallow networks use only a few layers to avoid the loss of semantic information; however, these networks lack robustness and generalization capabilities. Thus, we use only 10 convolution layers in each encoder and decoder and confine the feature map size to 31 × 31. Figure 5 shows a detailed diagram of the proposed R-SNN, and Table 2 lists the layer-wise details of the proposed R-SNN.

### 3.4. Loss Function

Loss functions are used during the training of the model to calculate the penalty of any deviation of the predicted output from the actual output. The partial derivatives of the loss function are calculated for each trainable weight of the model, and these weights are adjusted to obtain a minimal loss. Various types of loss functions have been adopted, such as Dice loss [45], focal loss [46], mean squared error or hinge loss [47], and log loss (cross-entropy loss) [48]. In the proposed R-SNN, we used cross-entropy loss because of its logarithmic function and probabilistic approach. In our nuclear segmentation task, ROIs are tiny and over- and under-segmentation can occur. Therefore, cross-entropy loss helps to avoid gradient saturation for extreme values, and the probabilistic approach penalizes both types of errors (over-segmented and under-segmented). In the cross-entropy loss, the output of the model is shown as a probability between 0 and 1, and its value increases as the predicted probability diverges from the actual label. This can be expressed by Equation (3).

(3) $$\mathrm{Loss}=-\frac{1}{M}\sum_{m=1}^{M}\left[y_{m}\cdot log\left(h_{\theta }\left(x_{m}\right)\right)+\left(1-y_{m}\right)\;\cdot \;log\left(1-h_{\theta }\left(x_{m}\right)\right)\right],$$

where *M*, *y_m_*, *x_m_*, and *h_θ_* are the number of training data, the target label for training data *m*, the input for training data *m*, and the model with weight *θ*, respectively. In cross-entropy loss, only the probability of a data point assigned to its corresponding ground-truth class is emphasized.

## 4. Experiments and Performance Analysis

This section presents the datasets, experimental hardware, and software specifications along with the evaluation criteria and performance analysis.

### 4.1. Datasets

In our experiments, we used two publicly available datasets of nuclear segmentation, namely, TCGA [29] and TNBC [32] datasets. The datasets are described in detail below.

#### 4.1.1. TCGA Dataset

**Figure 6:**
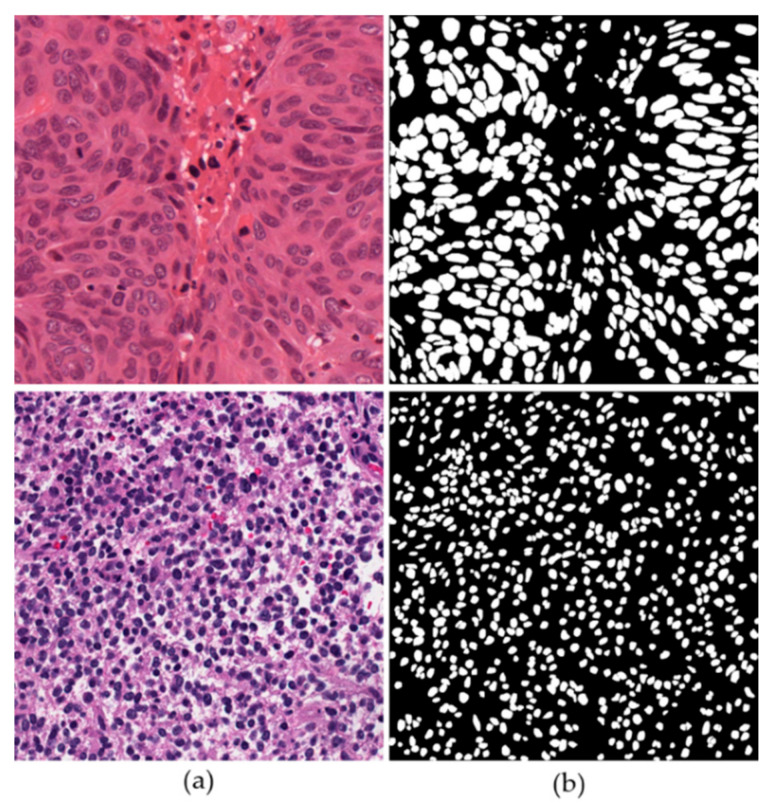
Sample images of breast (**upper**) and kidney (**lower**) cancers from TCGA dataset: (**a**) image and (**b**) ground truth.

TCGA is a publicly funded project that aims to create an atlas of cancer genomic profiles. To date, over 20,000 cases of 33 cancer types have been analyzed by TCGA researchers. The major objective is to provide publicly available datasets [49]. Kumar et al. selected 44 WSIs of multiple organs and generated ground truths for the nuclear segmentation task [29]. Each WSI is cropped from a nuclear-dense area to a sub-image of size 1000 × 1000 at a magnification of 40×. There are 44 images in total, among which the provider predetermined 30 images for training and 14 for testing without a validation set. We followed the same scheme for a fair comparison of previous researches. Nine organs, i.e., breast, liver, kidney, prostate, bladder, colon, stomach, lung, and brain, are represented in this dataset. A multi-organ nucleus segmentation challenge (MoNuSeg 2018) was also successfully organized using this dataset at the International Conference on Medical Image Computing and Computer-Assisted Intervention (MICCAI 2018) [50]. The objective of this challenge was to develop generalized nucleus segmentation techniques. Table 3 summarizes the composition of TCGA dataset, and Figure 6 shows sample images from TCGA dataset.

#### 4.1.2. TNBC Dataset

**Figure 7:**
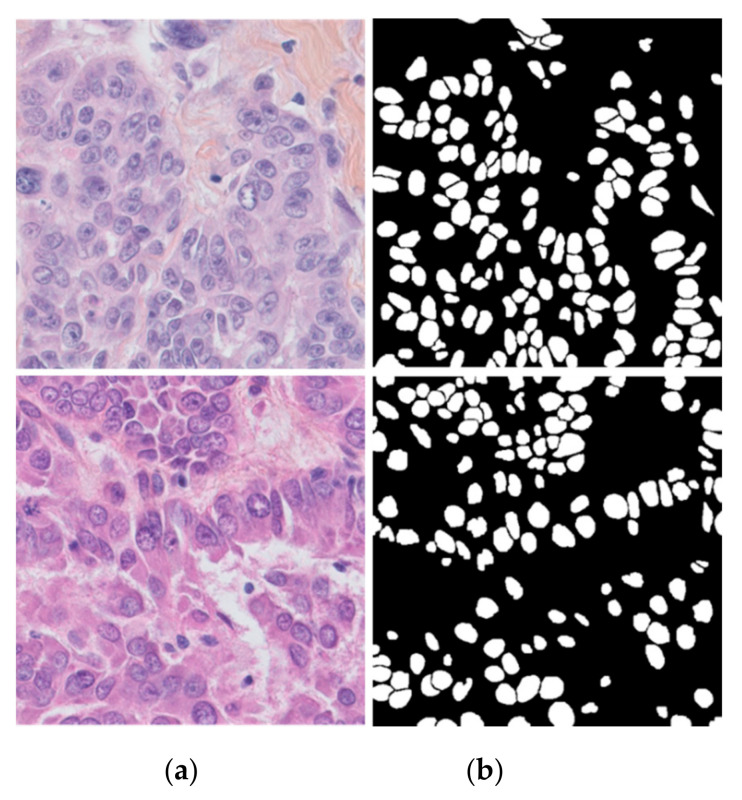
Sample images of breast cancer of the TNBC dataset: (**a**) image and (**b**) ground truth.

The TNBC dataset was presented by Naylor et al. along with their nuclear segmentation technique for breast cancer histopathology images [32]. TNBC is a type of breast cancer in which the cancer cells do not have estrogen or progesterone receptors and do not make too much of the protein called HER2. This type of breast cancer spreads faster than other invasive breast cancers [51]. The WSI images of 11 patients were randomly selected from an unpublished TNBC patient database and cropped at multiple random positions at a magnification of 40× and a size of 512 × 512 to obtain this dataset. Then, 3–7 images having diverse and complex nuclei were selected. There were a total of 50 images from 11 patients. The leave-one-patient-out scheme was used for experiments. In every experiment, data from eight patients were used for training, data from two patients were used for validation, and the remaining data from one patient were used for testing. Figure 7 shows the sample images from the TNBC dataset.

### 4.2. Data Augmentation

Diverse variations usually exist even within the patterns of the same class of training and testing data. Training with a large amount of data is necessary to develop a robust and accurate model. However, the collection of large amounts of data is difficult in the medical domain. Data augmentation can be used to solve the problem of limited data. To this end, the original training data are transformed using various transformations, and new training data are generated and added to the original data. In our study, TCGA dataset has 30 images for training, whereas the TNBC dataset has 50 images for both training and testing. For data augmentation, translation and cropping are employed first, followed by horizontal and vertical flipping. Then, random translation is employed on the *x*- and *y*-axes. In the proposed work, we applied offline augmentation to increase the quantity of training data for successful training. That is, data augmentation was not performed on each epoch of training. Instead, it was done in advance before training, and we started the training with the already augmented training data.

**Figure 8:**
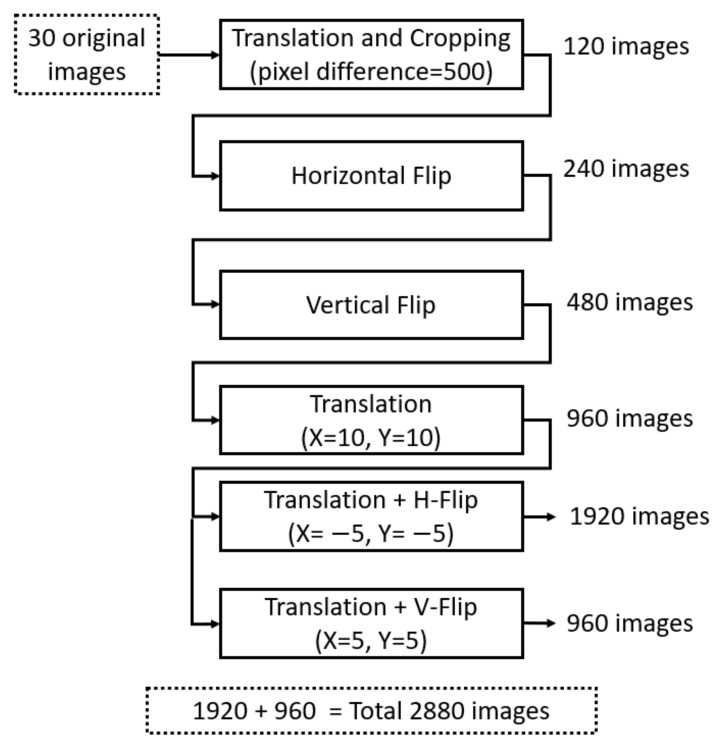
Data augmentation scheme used in the proposed technique. H-Flip and V-Flip indicate horizontal and vertical flipping, respectively.

In the case of TCGA dataset, an image of size 1000 × 1000 was cropped to 500 × 500 with a pixel difference of 500 and, thus, 120 images were produced. Horizontal flipping was then performed, yielding 120 additional images. Thus, these 240 images were further vertically flipped, producing 480 images. Then, translation at *x* = 10 and *y* = 10 was performed, followed by cropping and resizing. This technique produced 480 additional images. From these procedures, 960 images were produced, which were further augmented by translation at *x* = −5 and *y* = −5, cropping, resizing, and horizontal flipping, yielding a total of 1920 images. Then, 960 additional images were produced using the same scheme by performing translation at *x* = 5 and *y* = 5. Then, the total number of training images was 2880 in TCGA dataset, which was sufficient for the successful training of our network. Table 4 and Figure 8 present detailed explanations of the data augmentation for TCGA dataset.

In the case of the TNBC dataset, 50 images were used for both training and testing using the leave-one-patient-out scheme. In the first step, a pixel difference of 10 was used for translation and cropping because the original image size was 512 × 512. The same schemes of data augmentation were then applied. As the leave-one-patient-out scheme was used in the experiments, an example of data augmentation used for the testing of patient 1 is presented in Table 4. A total of 43 images were augmented to 2064 images using the same data augmentation as for TCGA dataset.

### 4.3. Experimental Setup and Training

#### 4.3.1. Experimental Setup

The proposed technique was implemented using MATLAB R2019a (MathWorks, Inc., Natick, MA, USA) [52] on a desktop computer operating with the Windows 10 operating system. The desktop computer included a central processing unit (CPU) with a 3.60 GHz Intel^®^ (Santa Clara, CA, USA) Core-i7-7700 [53], 16 GB random access memory (RAM), and an NVIDIA GeForce GTX 1070 graphic processing unit (GPU) with 8 GB GPU memory [54].

#### 4.3.2. Training

**Figure 9:**
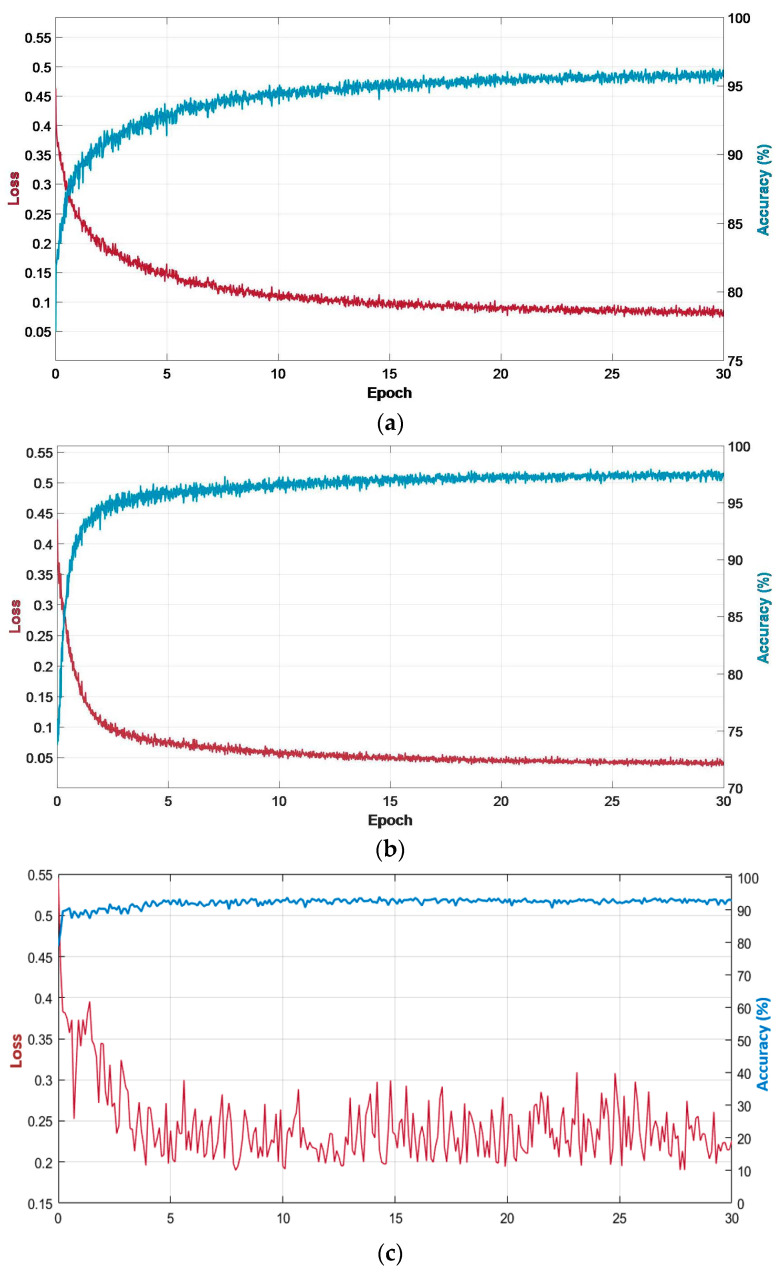
Training and validation loss and accuracy curves of the proposed R-SNN. Training loss and accuracy curves with (**a**) TCGA dataset and (**b**) the TNBC dataset. Validation loss and accuracy curves with (**c**) the TNBC dataset.

The proposed R-SNN was trained from scratch without prior weight initialization. Random weights were assigned at the beginning of training. The training parameters were maintained the same for both datasets. In TCGA dataset, the training images were fixed by the providers, whereas the leave-one-patient-out scheme was used for training with the TNBC dataset. We used the Adam optimizer because it is considered robust to the values of hyperparameters and can handle sparse gradients on challenging problems such as nuclear segmentation [55]. The other training parameters were an initial learning rate of 0.0001, a mini-batch size of 4, an L2 regularization of 0.0005, and a gradient threshold of 8. The best configuration for these parameters was experimentally found with training data as a function of training accuracy. During training, the proposed R-SNN converged faster in only 30 epochs owing to the spatial information transfer from the encoder to the decoder through residual connectivity and fewer convolution layers. Training for more epochs did not improve the performance of the network. Figure 9 presents the training accuracy and loss curves during the training of the proposed R-SNN on TCGA and TNBC datasets. Figure 9a–c show the training accuracy and loss curves for TCGA and TNBC datasets, and the validation accuracy and loss for TNBC dataset, respectively. It can be observed from the convergence of curves in these figures that training was successfully performed for both datasets.

### 4.4. Performance Evaluation of the Proposed Method

#### 4.4.1. Performance Evaluation Metric

The evaluation criteria for nuclear segmentation methods need to penalize both object-level and pixel-level errors. Therefore, we used two different evaluation metrics: object-level and pixel-level metrics. For object-level evaluation, the F1-measure was used [56], whereas Dice’s coefficient (DC) [57] and aggregated Jaccard index (AJI) [29] were used for pixel-level evaluations. We used the threshold of true positives (TP) at the object level as 50% as in most previous studies. The F1-measure was used only for object-level evaluations in all tables presented in Section 4.4.2 and Section 4.4.3, and there is no table where the F-measure was used for pixel-level evaluation in our manuscript. On the other hand, DC and AJI were used only for pixel-level evaluations in all tables presented in Section 4.4.2 and Section 4.4.3.

The F1-measure is the harmonic average of precision and recall. The F1-measure evaluates precision and recall simultaneously. If the ground-truth objects are represented by Gi and segmented objects by S_j_ (i and j represent indices), the F1-measure, precision, and recall are evaluated by TP, false positives (FP), and false negatives (FN). TP is the count of all the ground-truth objects G_i_ with the correctly segmented objects S_j_. FP is the count of all the incorrectly segmented objects S_j_ that are not actually the ground-truth objects G_i_. FN is the count of incorrectly unsegmented objects S_j_ that are the ground-truth objects G_i_. In terms of these values, the F1-measure, precision, and recall are expressed by Equations (4)–(6), respectively.

(4) $$F1-\mathrm{measure}=\frac{2\mathrm{TP}}{2\mathrm{TP}+\mathrm{FP}+\mathrm{FN}},$$

(5) $$\mathrm{Precision}=\frac{\mathrm{TP}}{\mathrm{TP}+\mathrm{FP}},$$

(6) $$\mathrm{Recall}=\frac{\mathrm{TP}}{\mathrm{TP}+\mathrm{FN}}.$$

The F1-measure does not consider pixel-level errors, and it cannot be used to evaluate over-segmentation and under-segmentation. Therefore, along with the F1-measure, Dice’s coefficient (DC) [57] and aggregated Jaccard index (AJI) [29] were also used. DC and AJI measure the quality of segmentation at the pixel level. If pixels of a ground-truth nucleus are represented by G_i_ and its associated segmented nucleus by S_i_, then DC can be expressed by Equation (7).

(7) $$\mathrm{DC}=2\;.\;\frac{\left|{\;G}_{i\;}\cap {\;S}_{j}\right|\;}{\left|G_{i\;}\right|+\left|S_{j}\right|}.$$

AJI was also used as an evaluation criterion along with the DC for pixel-level evaluation. AJI is the extension of the Jaccard index and is defined as follows:

(8) $$\mathrm{AJI}=\frac{\sum_{i=1}^{L}\left|G_{i}\cap P_{i}\right|\;}{\sum_{i=1}^{L}\left|G_{i}\cap P_{i}\right|+\sum_{i\epsilon \;\mathrm{rest}}^{L}\left|P_{i}\right|\prime \;}.$$

P_i_ is the predicted nucleus that maximizes the Jaccard index with the ground-truth nucleus G_i_, and the remainder refers to the collection of P_i_ with no match. AJI reflects the proportion between the common region of matched elements and the segmented results. Any imprecise segmentation, whether under- or over-segmentation, will lead to a decrease in AJI.

#### 4.4.2. Ablation Study

An ablation study was conducted in which the effects on the performance of a method are analyzed by removing or adding a certain feature or module. We studied the effects of data augmentation, stain normalization, residual skip connectivity, number of layers, and robustness of the network. Data augmentation was used to solve the problem of limited data for training of deep learning models. The original training data were transformed using various transformations, and new training data were generated and added to the original data. Experimental results show that data augmentation played a crucial role in our proposed method. It can be seen in Table 5 that the proposed network had higher accuracy when trained with augmented data than the case with no data augmentation.

After data augmentation, we studied the role of stain normalization in our proposed method. Stain normalization is important in histopathology images because the inconsistencies created due to various factors are removed, which boosts the performance of the model. Two experiments were performed with the same parameters and environmental setup to confirm the above. In experiment 1, stain-normalized data were used, whereas stain normalization was not employed in experiment 2. Experimental results show that the accuracy with stain normalization was higher than that without stain normalization, as presented in Table 6, which confirms the necessity of stain normalization for accuracy enhancement.

In the next set of experiments, we studied the residual connections and number of layers in the network. The proposed network with and without residual connectivity (concatenation and addition) and different numbers of layers are studied. The proposed network used 91 layers including 26 convolution layers in the encoder and decoder parts of the network. Residual connections (concatenation and addition) were added and, after multiple experiments, we found that the proposed network-RC (addition) had a higher accuracy than the proposed network (no skip connections) and proposed network-RC (concatenation). Table 7 presents the results of these experiments. Next, we tested the performance of our proposed network by reducing and increasing the number of layers. The number of layers is directly proportional to the computation cost of the model. It was found that the proposed network-RL which used 75 layers had a higher accuracy than the other networks. Results can be found in Table 7. The combined effect of residual connections and reduced network produced better results than the other methods in terms of the F1-measure, although the AJI and DC of the proposed network-RC + RL were similar to those of the proposed network-RL.

The robustness toward various image sizes of the proposed network was studied by resizing the test images to 500 × 500, 1500 × 1500, and 2000 × 2000 pixels. Table 8 presents the experimental results. It can be seen that the performance with the test image of 1000 × 1000 pixels was superior to the other cases. When we magnified our test image size to 2000 × 2000 pixels, background noise was also boosted, resulting in a reduction in performance. The performance on 1500 × 1500 pixel images was better than that on 2000 × 2000 pixel images due to the slight increase in nuclear size and background region. Contrary to this, the results with 500 × 500 pixel images were better than those with the 2000 × 2000 and 1500 × 1500 pixel images due to the reduction in background noise, despite the reduction in nuclear size.

We also tested other loss functions such as Dice loss [45] and focal loss [46], and the experimental results proved that the accuracy with cross-entropy loss was higher than that with other loss functions, as shown in Table 9.

#### 4.4.3. Comparisons with State-of-the-Art Methods

**Figure 10:**
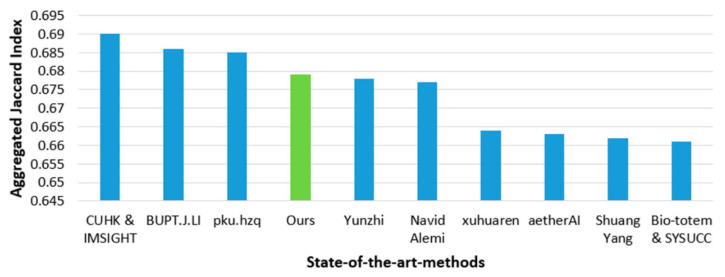
MICCAI 2018 leaderboard results in comparison to the proposed method.

In this section, we compare the performance of the proposed method with that of state-of-the-art methods for nuclear segmentation. For a fair comparison, the training and testing data along with the evaluation criteria were the same as those used for the state-of-the-art methods. As shown in Table 10 and Table 11, the proposed method had higher accuracy than the existing techniques because of the adoption of stain normalization and data augmentation, along with the R-SNN. Furthermore, we also present TCGA dataset challenge (MICCAI 2018) [50] challenge results in comparison to our proposed method. The leaderboard of MICCAI 2018 is presented in the Figure 10. The top scorers (CUHK and IMSIGHT) achieved outstanding performance by using contour information aggregation-based networks with extensive data augmentation. Smaller nuclei were missed, while larger nuclei were over-segmented by this technique. The second-best scorer (BUPT.J.LI) proposed a deep layer aggregation-based network [58] using color normalization as a preprocessing. Unwanted overly smooth nuclei boundaries were produced by this method. The third-best scorer (pku.hzq) used a combined U-Net [59] and Mask R-CNN [60]. Although the online evaluation of the MICCAI 2018 challenge is closed, test data are publicly available. After testing with the test data from the MICCAI 2018 challenge, we found that our proposed method was ranked fourth on the leaderboard. However, we used a shallower model compared to those ranked above, confirming the lower number of training parameters and system complexity of our model.

#### 4.4.4. Correct and Incorrect Detection Cases Using the Proposed Method

**Figure 11:**
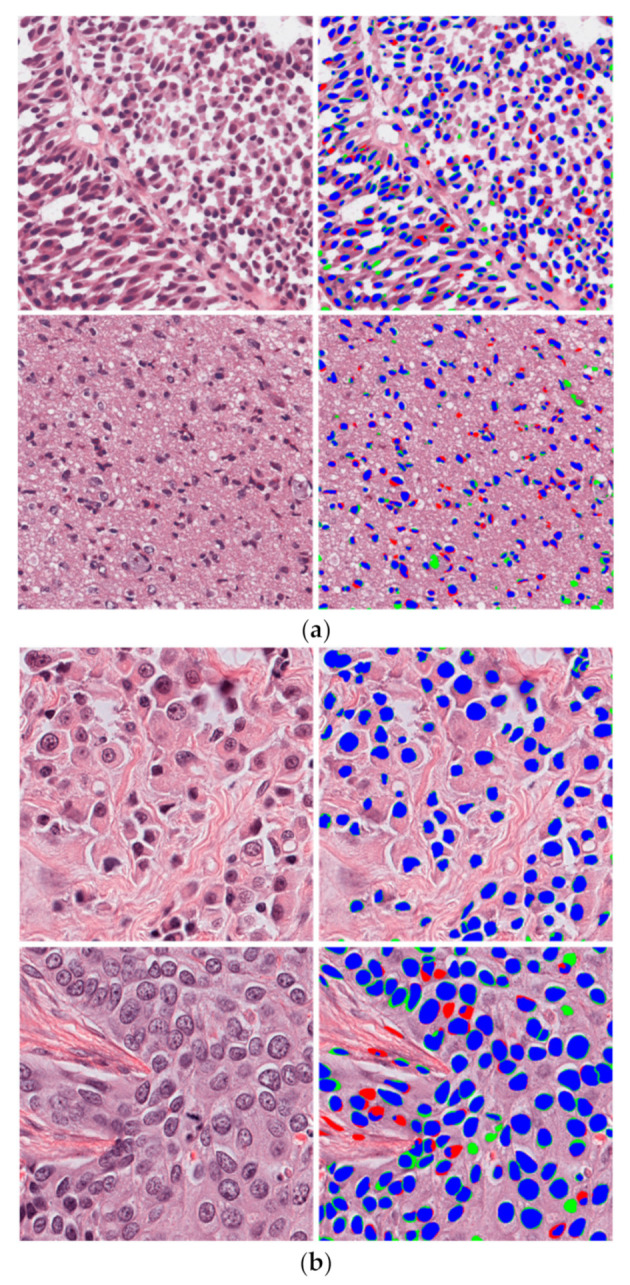
Examples of good segmentation performance of the proposed method on test images from (**a**) TCGA dataset and (**b**) the TNBC dataset. Blue, red, and green regions represent TP, FN, and FP, respectively.

**Figure 12:**
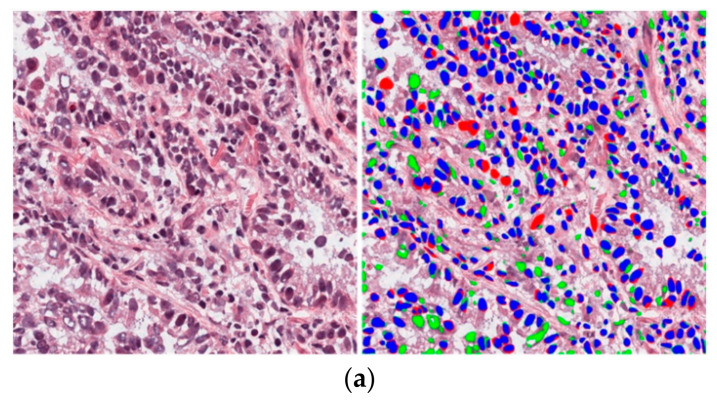

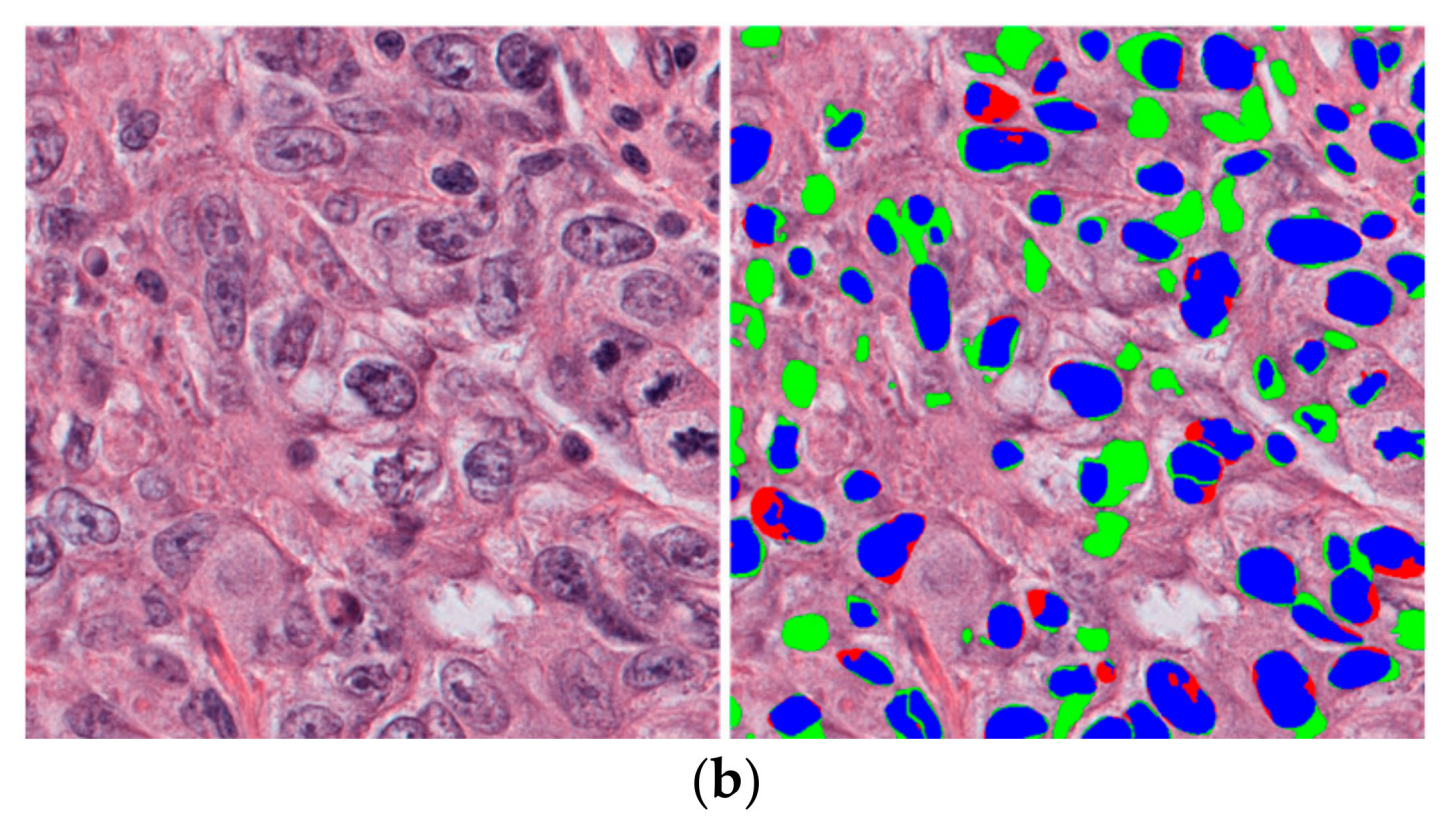
Examples of bad segmentation performance of the proposed method on test images from (**a**) TCGA dataset and (**b**) the TNBC dataset. Blue, red, and green regions represent TP, FN, and FP, respectively.

The proposed method was tested on a diverse set of images from both datasets. Testing images were obtained from nine organs: brain, breast, kidney, liver, prostate, bladder, colon, stomach, and lungs. As the composition of tissues in each organ is different, segmentation results differed according to the organ. Nevertheless, we obtained a good segmentation performance, as shown in Figure 11. Figure 11a,b show the images taken from TCGA and TNBC datasets, respectively. However, an incorrect segmentation performance was also obtained in a few cases where the nuclei were overlaid by the background and a high overlap existed among nuclei, as shown in Figure 12.

## 5. Discussion

**Figure 13:**
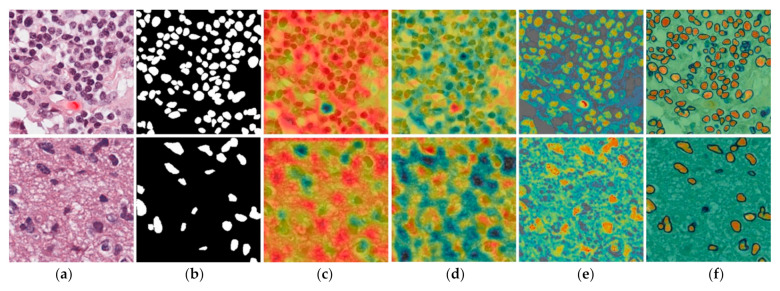
Input and ground-truth images, as well as Grad-CAM activation maps extracted from the intermediate layers of the proposed network. Two examples are shown in the first and second rows: (**a**) input image, (**b**) ground-truth image, (**c**) EConvBR-1_1 layer, (**d**) EConvBR-4_3 layer, (**e**) DConvBR-4_2 layer, and (**f**) DConvBR-1_2 layer of the network, as presented in the Table 2.

Deep learning models are considered as black boxes because there is no explanation behind the prediction. It is difficult to determine how the network extracts the important features in the input image, which neurons are activated during processing, and how the network arrived at its final output. A visual explanation of the prediction plays a key role in building trust in intelligent systems. Gradient-weighted class activation maps (Grad-CAM) [61] were proposed for visual analysis of deep learning networks. In this technique, activation maps along the feature channel are extracted and averaged to be presented as a single image. This single Grad-CAM image is represented in a pseudo color scheme, in which the red color indicates the maximum value and the blue color shows the minimum value of an intensity to activation. In this way, we obtain a coarse localization map which highlights the regions in the image for a prediction. Figure 13a,b present the input and ground-truth images, respectively, and Figure 13c–f show the Grad-CAM images extracted from various layers of our R-SNN. In Figure 13c, the Grad-CAM image was extracted from the first layer (EConvBR-1_1), and, in Figure 13d, it was extracted from the last convolution layer (EConvBR-4_3) of the encoder block. Figure 13e shows the Grad-CAM image extracted from the initial layer (DConvBR-4_2) of the decoder block. Figure 13f presents the Grad-CAM image from the DConvBR-1_2 layer of the decoder block, and it can be observed that nuclear areas were highlighted. Nevertheless, these could also be taken from the other layers of Table 2. Activation maps allow a visual explanation of the network’s decision making. During the training process, networks can learn and draw activation maps from noisy objects or background. We developed activation maps visually discriminate the ROIs contributing most during training of the network. The F1-score gives the final result of segmentation, whereas activation maps provide visual discriminative features, as well as the activations contributing most to the training process.

In conclusion, it is clear from these activation maps that our proposed network is not biased and was successfully trained to differentiate between the background and nucleus.

Nuclear segmentation in the histopathology images of cancer plays a key role in its diagnosis and prognosis. AI-based segmentation is fast and robust and shows better segmentation results than handcrafted-feature-based segmentation. It also saves the time and effort required for the inspection of histopathology images by humans under high-resolution microscopes. In this article, we proposed an AI-based nuclear segmentation technique in which an image was first stain-normalized to remove inconsistencies, followed by nuclear segmentation through the novel R-SNN. Experiments on publicly available datasets proved that the proposed method showed better accuracy compared with state-of-the-art nuclear segmentation methods. Some of the key observations derived from this work are as follows:

- Stain normalization plays a key role in the classification, detection, and segmentation of histopathology images because it removes the inconsistencies caused by the staining and environmental factors. The performance of deep-learning models was also improved, as presented in Table 6, where the experiments performed with stain normalization showed higher accuracy than those without stain normalization.
- Shallow networks lack generalization capabilities; therefore, deep networks are mostly developed. However, in the case of applications related to histopathology images, a severe vanishing gradient problem mostly occurs because the ROIs are usually tiny and, thus, may vanish due to successive convolution operations. In our proposed technique, we confined the feature map size to 31 × 31, which positively influenced the performance of our segmentation model. The performance of SegNet was lower than that of the proposed technique because the final feature map size of the encoder was 7 × 7 in SegNet, indicating that important information may be lost in successive convolution operations.
- The residual skip connections from the encoder to the decoder of the proposed R-SNN empowered feature representation, which enabled the network to perform well despite having only a few layers of convolution. In the proposed technique, only 10 convolution layers were used in the encoder. However, the proposed model showed higher accuracy than the state-of-the-art models because the residual skip connections retained important information helpful for nuclear segmentation.
- AI-based applications can be applied to digital pathology because of their good generalization capabilities, high performance, and short inference time. Experiments on two datasets—TCGA dataset containing images from breast, kidney, liver, prostate, bladder, colon, stomach, lung, and brain, and the TNBC dataset containing breast cancer images—proved that AI-based applications have a good generalization capability.
- Accurate, robust, and computationally inexpensive AI-based methods play a very important role in boosting the confidence level of pathologists toward AI. In this regard, the proposed method can be adopted for real-time applications owing to its good performance, robustness, and low computational cost.
- Despite the above, our proposed work had a few limitations. First, an intensive task of stain normalization was performed, which could cause high computational complexity. Second, neighboring nuclei were difficult to separate and were considered a single object. Third, many applications involve whole-slide images, which are much larger than 1000 × 1000 pixels. The extension of our research to whole-slide image processing across different magnifications is required as future work.

Pathologists and AI-based methods can work together in cancer diagnosis and prognosis. A simple mistake in cancer diagnosis can have disastrous consequences for patients. Therefore, AI can be adopted to assist pathologists as a second opinion. Moreover, AI-based methods can segment ROIs, indicate positive cases, and reduce the time taken during clinical applications.

## 6. Conclusions

In this study, we aimed to develop a semantic segmentation method for nuclear segmentation in multi-organ histopathology images. Segmented nuclei play a key role in cancer diagnosis and prognosis. Histopathology images from two publicly available datasets—TCGA and TNBC—were used in this study. In the proposed method, a histopathology image was stain-normalized and input to the trained model, which output segmented images with nuclei. The proposed R-SNN maintains crucial features by using the residual connectivity from the encoder to the decoder, and it also uses only a few layers, which reduces the computational cost of the model. The selection of a good stain normalization technique, the effective use of residual connections to avoid information loss, and the use of only a few layers to reduce the computational cost yielded outstanding results. Thus, our nuclear segmentation method is robust and superior to the state-of-the-art methods. We expect that this study will contribute to the development of computational pathology software for research and clinical use and enhance the impact of computational pathology.

In the future, we aim to increase the generalization capability of the proposed method by testing it on more datasets with larger whole-slide images, as well as improve the segmentation performance by using other convolution types such as separable, dilated, and deformable convolutions. Furthermore, novel stain normalization and data augmentation methods will be studied.

## Figures and Tables

**Table 1:** Summarized comparisons of the proposed and state-of-the-art techniques for nuclear segmentation.

Type	Techniques	Strength	Weakness
Handcrafted feature-based	Marker-controlled watershed segmentation, and combination of mean shift and the Kalman filter for the tracking of nuclei [21]	Superior performance to other techniques such as k-means clustering	-Long inference time -Optimization of marking region is required.
	Segmentation based on the Hough transform and active model [22]	-Robust to noisy data -Can handle missing information -Can easily adapt to different shapes	-Computationally complex and expensive, and has a low accuracy -Inability to separate connecting objects
	Active contour and shape model with the integration of region, boundary, and shape information [24]	Autonomous and self-adaptation can track objects in both temporal and spatial directions	-Need for an initial counter -Can become stuck in local minima -Computationally expensive and has a long runtime -Cannot manage intensity inhomogeneity effectively
	Marker-controlled watershed segmentation, snake model, and SVM classifier [20]	-Resulting boundaries of the objects correspond to contours -Postprocessing not required	-Excessive over-segmentation and requires the optimization of marker -Long inference time
Deep feature-based	Mask-RCNN [28]	Good for instance segmentation	High computational cost of region proposals
	Three-class CNN [29]	Simple structure	Uses many parameters due to fully connected layers
	PangNet, FCN, DeconvNet, and ensemble classification [32]	Good for jointly segmented nuclei	Postprocessing overhead and low accuracy
	Two-stage learning using U-Net and DLA [36]	High accuracy by considering nuclear segmentation as a three-class problem	Computationally expensive due to two stages and multiple networks
	Multilevel information aggregation using task-specific decoders and novel smooth truncated loss [37]	Good generalization capability because the network focuses on learning from reliable and informative samples	Computationally expensive due to multiple decoder networks
	U-Net-based classification and regression in two sequential stages [38]	-Good performance for the prediction of the pixels of the border -Images of different sizes can be used as input due to the absence of a dense layer in U-Net	Postprocessing overhead and requires many learnable parameters
	U-Net variant [39]	-Inception module captures detailed information	Postprocessing overhead
	U-Net variant with group-equivariant convolution and upsampling [40]	-Easy training on multiple GPUs and possibility of model parallelization -Can learn powerful representations based on symmetry pattern	-Postprocessing overhead and requires many learnable parameters -Low performance due to the lack of meaningful relationships based on relative positions, orientations, and scales
	R-SNN (the proposed method)	Robust segmentation with fewer trainable parameters without postprocessing	Stain normalization as preprocessing

**Table 2:** Layer-wise details of the proposed R-SNN (EB-n = encoder block-n, *RC-n* = residual connection-n, EConvBR = encoder block convolution + batch normalization + ReLU, DConvBR = decoder convolution + batch normalization + ReLU, DB-n = decoder block-n, *Add-n* = element-wise addition).

Block	Name/Size	Number of Filters	Output Feature Map Size (Width × Height × Number of Channels)	Number of Trainable Parameters
EB-1	EConvBR-1_1/3 × 3 × 3 *To decoder (RC-1)*	64	500 × 500 × 64	1792 + 128
	EConvBR-1_2/3 × 3 × 64	64		36,928 + 128
Pooling-1	Pool-1/2 × 2	-	250 × 250 × 64	-
EB-2	EConvBR-2_1/3 × 3 × 64 *To decoder (RC-2)*	128	250 × 250 × 128	73,856 + 256
	EConBR-2_2/3 × 3 × 128	128		147,584 + 256
Pooling-2	Pool-2/2 × 2	-	125 × 125 × 128	-
EB-3	EConvBR-3_1/3 × 3 × 128 *To decoder (RC-3)*	256	125 × 125 × 256	295,168 + 512
	EConvBR-3_2/3 × 3 × 256	256		590,080 + 512
	EConvBR-3_3/3 × 3 × 256	256		590,080 + 512
Pooling-3	Pool-3/2×2	-	62 × 62 × 256	-
EB-4	EConvBR-4_1/3 × 3 × 256 *To decoder (RC-4)*	512	62 × 62 × 512	1,180,160 + 1024
	EConvBR-4_2/3 × 3 × 512	512		2,359,808 + 1024
	EConvBR-4_3/3 × 3 × 512	512		2,359,808 + 1024
Pooling-4	Pool-4/2 × 2	-	31 × 31 × 512	-
Unpooling-4	Unpool-4	-	62 × 62 × 512	-
DB-4	DConvBR-4_3/3 × 3 × 512	512		2,359,808 + 1024
	DConvBR-4_2/3 × 3 × 512	512		2,359,808 + 1024
	*Add-4 (DConvBR-4_2 + RC-4)*	-		-
	DConvBR-4_1/3 × 3 × 512	256	62 × 62 × 256	1,179,904 + 512
Unpooling-3	Unpool-3	-	125 × 125 × 256	-
DB-3	DConvBR-3_3/3 × 3 × 256	256		590,080 + 512
	DConvBR-3_2/3 × 3 × 256	256		590,080 + 512
	*Add-3 (DConvBR-3_2 + RC-3)*	-		-
	DConvBR-3_1/3 × 3 × 256	128	125 × 125 × 128	295040 + 256
Unpooling-2	Unpool-2	-	250 × 250 × 128	-
DB-2	DConvBR-2_2/3 × 3 × 128	128		147,584 + 256
	*Add-2 (DConvBR-2_2 + RC-2)*	-		-
	DConvBR-2_1/3 × 3 × 128	64	250 × 250 × 64	73,792 + 128
Unpooling-1	Unpool-1	-	500 × 500 × 64	-
DB-1	DConvBR-1_2/3 × 3 × 64	64		36,928 + 128
	*Add-1 (DConvBR-1_2 + RC-1)*	-		-
Output	DConvBR-1_1/3 × 3 × 64	2	500 × 500 × 2	1154 + 4

**Table 3:** Number of images of TCGA dataset divided into training and testing data. “-” indicates not given.

Data	Organ									
	Total	Breast	Kidney	Liver	Prostate	Bladder	Colon	Stomach	Lung	Brain
Training	30	6	6	6	6	2	2	2	-	-
Testing	14	2	3	-	2	2	1	-	2	2

**Table 4:** Number of images produced in data augmentation.

Dataset	Original Images	Data Augmentation				Total
		Translation and Cropping	Horizontal Flipping	Vertical Flipping	Translation, Cropping, and Resizing	
TCGA	30	120	120	240	2400	2880
TNBC	43	172	172	344	1376	2064

**Table 5:** Ablation study on the effect of data augmentation on the performance on TCGA dataset. AJI and DC were used for pixel-level evaluations, whereas the F1-measure was used for object-level evaluations.

Methods	AJI	DC	F1-Measure
No data augmentation	0.5546	0.7120	0.6845
With data augmentation	0.6420	0.7749	0.8165

**Table 6:** Ablation study on the effect of stain normalization on the performance on TCGA dataset. AJI and DC were used for pixel-level evaluations, whereas the F1-measure was used for object-level evaluations.

Methods	AJI	DC	F1-Measure
Without normalization	0.6420	0.7749	0.8165
With normalization	0.6794	0.8084	0.8547

**Table 7:** Ablation study of the proposed technique with or without RC and Rl on TCGA dataset. AJI and DC were used for pixel-level evaluations, whereas the F1-measure was used for object-level evaluations.

Technique	AJI	DC	F1-Measure	Number of Parameters
Proposed Network (no skip connections)	0.6540	0.7902	0.7617	29,444,162
Proposed Network-RC (Concatenation)	0.6704	0.8020	0.8161	29,444,162
Proposed Network-RC (Addition)	0.6731	0.8039	0.8274	29,444,162
Proposed Network-RL	0.6738	0.8067	0.8342	15,279,174
Proposed Network-RC + RL	0.6794	0.8084	0.8547	15,279,174

**Table 8:** Ablation study of the proposed technique with different magnification of images of TCGA dataset. AJI and DC were used for pixel-level evaluations, whereas the F1-measure was used for object-level evaluations.

Size of Input Image	AJI	DC	F1-Measure
2000 × 2000	0.5410	0.7004	0.5996
1500 × 1500	0.6185	0.7633	0.7452
1000 × 1000	0.6794	0.8084	0.8547
500 × 500	0.5955	0.7453	0.7977

**Table 9:** Comparative accuracy of the proposed method using difference loss functions. AJI and DC were used for pixel-level evaluations, whereas the F1-measure was used for object-level evaluations.

Loss Function	AJI	DC	F1-Measure
Dice loss [45]	0.6764	0.8063	0.8475
Focal loss [46]	0.6726	0.8035	0.8317
Cross-entropy loss	0.6794	0.8084	0.8547

**Table 10:** Comparative accuracy of the proposed method and the state-of-the-art methods on TCGA dataset (“–” indicates not reported). AJI and DC were used for pixel-level evaluations, whereas the F1-measure was used for object-level evaluations.

Methods	AJI	DC	F1-Measure
Cell profiler [29,30]	0.1232	0.5974	0.4046
Fiji [29,31]	0.2733	0.6493	0.6649
Kumar et al. [29]	0.5083	0.7623	0.8267
Kang et al. [36]	0.5895	–	0.8079
Zhou et al. [37]	0.6306	–	0.8458
Mahbod et al. [38]	0.5687	0.7939	0.8267
Zeng et al. [39]	0.5635	0.8008	0.8278
Chidester et al. [40]	0.6291	0.7980	0.8490
Proposed method	0.6794	0.8084	0.8547

**Table 11:** Comparative accuracy of the proposed method and the state-of-the-art methods on the TNBC dataset. AJI and DC were used for pixel-level evaluations, whereas precision, recall, and F1-measure were used for object-level evaluations.

Methods	AJI	DC	Precision	Recall	F1-Measure
PangNet [32,33]	–	–	0.814	0.655	0.676
DeconvNet [32,35]	–	–	0.864	0.773	0.805
FCN [32,34]	–	–	0.823	0.752	0.763
Ensemble [32]	–	–	0.741	0.900	0.802
Kang et al. [36]	0.611		0.826	0.833	0.829
Proposed method	0.7332	0.8441	0.8352	0.8306	0.8329

## Data Availability

Not applicable.
